# Prosthetic valve endocarditis caused by *Pseudomonas luteola*

**DOI:** 10.1186/1471-2334-5-82

**Published:** 2005-10-12

**Authors:** Jean-Paul Casalta, Pierre-Edouard Fournier, Gilbert Habib, Alberto Riberi, Didier Raoult

**Affiliations:** 1 Laboratoire de Microbiologie, IFR 48, Centre Hospitalier-Universitaire de La Timone,264 rue Saint Pierre, Marseille, France; 2 Service de Cardiologie, Centre Hospitalier Universitaire de La Timone, Marseille, France; 3 Service de Chirurgie Cardiaque, Centre Hospitalier Universitaire de La Timone, Marseille, France

## Abstract

**Background:**

*Pseudomonas luteola *has been recognized as an uncommon cause of bacteremia and of infections in patients with underlying medical disorders

**Case presentation:**

We isolated *P. luteola *from blood cutures in a patient with prosthetic valve endocarditis developed 16 months after cardiac surgery.

**Conclusion:**

*P. luteola *is a rare opportunistic agent, with a propensity of infecting valvular prostheses.

## Background

*Pseudomonas luteola *(*P. luteola*) is an aerobic, Gram-negative rod with a distinctive yellow to orange pigment. After 48 hours of incubation, colonies are typically rough or wrinkled. The organism is non-fermentative, oxidase-negative, catalase-positive, and grows on MacConkey agar [1]. The organism was originally named *P. luteola*. On the basis of low levels of DNA- DNA hybridization, it was subsequently reclassified as *Chryseomonas luteola *[1]. Anzai *et al. *[2], in an analysis of 16S rDNA sequences of these organisms, has suggested that genus names *Chryseomonas, Flavimonas *and *Pseudomonas *were synonymous. Consequently, they concluded that the names *P. luteola *and *Pseudomonas oryzihabitans *should be used. The normal habitat of *P. luteola *is unclear, although it belongs to a group of bacteria normally found in water, soil, and other damp environments [3,4]. Reported human infections are rare.

## Case presentation

In July 2003, a 53-year-old man was admitted to the Timone hospital in Marseilles, France, presenting with clinical signs of acute endocarditis. He had a fever of 39°C that lasted for two weeks, anorexia, a weight loss of 7 kg since December 2002, a stroke with intracranial haemorrhage, and femoral arterial emboli. He had had an aortic replacement by a bioprosthesis in March 2002 for aortic insufficiency. In February 2003, the patient was hospitalized for undulating fever (38.5°C) that had lasted for the previous 3 months. The transeosophageal echocardiography showed neither valvular dysfunction nor vegetation. Six blood cultures were negative. The patient was treated with amoxicillin (1 g twice a day orally) for 8 days. The fever decreased but persisted at a level of 37.8°C. In July 2003, upon his admission, the echocardiography (multiplane transesophageal echocardiography) showed a vegetation on the aortic bioprothesis valve measuring 30 mm at its maximum, and a grade IV valvular regurgitation. The white blood cell count was of 14.36 × 10^9^/L (92.7% polymorphonuclears, 5.0% lymphocytes, 2.3% monocytes), the haemoglobin level was of 90 g/L, the erythrocyte sedimentation rate (ESR) was of 50 mm/h (first hour), the C reactive protein level was of 208 mg/l. No rheumatoid factor was detected. All three aerobic blood cultures, as well as the removed femoral arterial thrombus yielded *Pseudomonas luteola *(*P. luteola*) within 48 h of culture. The microorganism was identified using both the API 20 E (Biomerieux,Marcy l'Etoile, France) and API 20 NE galeries (Biomerieux). The identification was confirmed by sequencing its 16S rDNA using the fD1 (AGAGTTTGATCCTGGCTCAG) and rP2 (ACGGCTACCTTGTTACGACTT) primers as previously described [2,5-7]. The nucleotide sequence (GenBank accession number AY574976) was compared with sequences available in GenBank using the BLAST version 2.2.9 software (National Center for Biotechnology Information) and showed 99.7% similarity with the 16S rDNA sequence of *P. luteola *(GenBank accession number D84002). The patient's isolate was susceptible to ampicillin, ureidopenicillin, third-generation cephalosporins, fluoroquinolones, and aminoglycosides. The patient was treated intravenously with ticarcillin + clavulanic acid (3 g five times per day) for 60 days, and gentamicin (210 mg once a day) for 15 days. The high dose of ticarcillin + clavulanic acid was justified by the cerebral involvement. In the course of antibiotic therapy, the fever resumed and the patient's condition improved. However, worsening aortic insufficiency led to the replacement of the aortic bioprothetic valve 76 days following admission. Macroscopic examination of the removed valve showed extensive aortic vegetation. Microscopic examination showed features typical of infectious endocarditis [8]. The bacterial culture of the valve was negative. The 16S rDNA PCR performed on valvular tissue was negative. Following cardiac surgery under extracorporeal circulation, the patient developed haemodynamic instability and renal insufficiency that required a prolonged hospitalization. The patient was released from hospital in February 2004, 7 months following admission.

Reported human *P. luteola *infections are rare. These have included a septicemia in a patient with systemic lupus erythematosus under corticosteroid therapy who developed haemorrhagic pancreatitis complicated by a pancreatic abscess [5]; one case of bacteremia in a previously healthy patient with granulomatous hepatitis [9]; a bacteremia in a patient with peritonitis [10]; and non-bacteremic cases of peritonitis associated with gangrenous appendititis [10] and continuous ambulatory peritoneal dialysis [11]. Bacteremia has also been reported in patients with indwelling vascular catheters [10-12]. Other clinical isolates have been recovered from the bone of a patient with a femur abscess [10]; from a patient with a subphrenic abscess [10]; from the cerebrospinal fluid and wounds of neurosurgical patients with dural grafts or bone flaps [13]; from an HIV-infected patient with invasive cutaneous infection [3]; and from a patient with facial cellulitis [11]. To the best of our knowledge, only two cases of endocarditis caused by *P. luteola *have been reported in patients with prosthetic cardiac valves [13,14]. These patients had developed fever and blood cultures grew *P. luteola *15 days [13] and 45 days [14], respectively, after cardiac surgery. In addition, one case of *P. luteola *septicemia has been described in a 5-month-old infant after open heart surgery for congenital cardiac disease [3]. Septicemia was diagnosed 8 days after surgery but no endocarditis was found. In the present case, as the patient did not undergo any invasive procedure between the 2002 valvular replacement and the onset of fever, we believe that he was infected during the initial cardiac surgery.

## Conclusion

Using the Duke endocarditis service criteria, our patient was classified as having a definite endocarditis (valvular histological examination confirmed the diagnosis of infectious endocarditis). The isolation of *P. luteola *in 3 blood cultures and in the arterial thrombus demonstrated its role as an etiologic agent. Including our patient, endocarditis with *P. luteola *has occurred in three patients who had undergone valvular replacement. This suggests that this organism is a rare opportunistic agent, with a propensity of infecting valvular prostheses.

## Competing interests

The author(s) declare that they have no competing interests.

## Authors' contributions

JPC isolated the microorganism, initiated the antibiotic therapy, and drafted the manuscript; PEF identified the microorganism and drafted the manuscript; GH performed the echocardiograms and drafted the manuscript; AR performed valvular surgery and drafted the manuscript; DR helped drafting the manuscript.

## Pre-publication history

The pre-publication history for this paper can be accessed here:


